# The influences of timing of urgent endoscopy in patients with acute variceal bleeding: a cohort study

**DOI:** 10.1186/s12876-022-02595-1

**Published:** 2022-12-08

**Authors:** Xiaohan Yan, Zhuyun Leng, Qinwei Xu, Zehua Zhang, Meidong Xu, Jingze Li

**Affiliations:** Endoscopy Center, Department of Gastroenterology, Shanghai East Hospital, Tongji University School of Medicine, No. 150 Jimo Road, Pudong New District, Shanghai, 200120 China

**Keywords:** Urgent endoscopy, Short-term mortality, Acute variceal bleeding

## Abstract

**Background:**

There has always been a debate on the optimal timing of endoscopy in patients with acute variceal bleeding (AVB).

**Objective:**

This study aimed to examine the relation between the timing of endoscopy and the short-term outcomes of patients with AVB.

**Methods:**

Patients with AVB who underwent endoscopy within 24 h after admission at our tertiary care center from 2014 to 2022 were evaluated retrospectively. The primary outcomes were the 6-week mortality and re-bleeding. The secondary outcomes included the total number of blood units transfused, the length of hospital stay, and the need for salvage therapy. We used Cox proportional hazards model to analyze the predictors of 6-week mortality in all patients as well as in those who were at high risk of further bleeding or death.

**Results:**

A total of 312 patients were enrolled. Among them, 170 patients (54.49%) underwent urgent endoscopy (< 6 h), and 142 patients (45.51%) underwent early endoscopy (6–24 h). There were no significant differences between the urgent-endoscopy group and the early-endoscopy group, regarding the 6-week mortality (16.47% vs. 10.56%; *P* value = 0.132) and 6-week re-bleeding rate (11.2% vs. 16.2%; *P* value = 0.196). In multivariate analysis, time to endoscopy was independent of 6-week mortality (*P* value = 0.170), but the time between the beginning of bleeding and endoscopy (within 12 h) was significantly associated with low 6-week mortality (OR: 0.16; 95% CI: 0.06–0.46; *P* value = 0.001). Time to endoscopy was still not associated with 6-week mortality in patients at high risk for further bleeding or death (Glasgow-Blatchford score ≥ 12, *n* = 138, *P* value = 0.902).

**Conclusions:**

Endoscopy performed within 6 h of admission, rather than within 6 to 24 h, did not improve six-week clinical outcomes in patients in stable condition with AVB and even those who were at high risk of further bleeding and death.

## Introduction

**Fig. 1:**
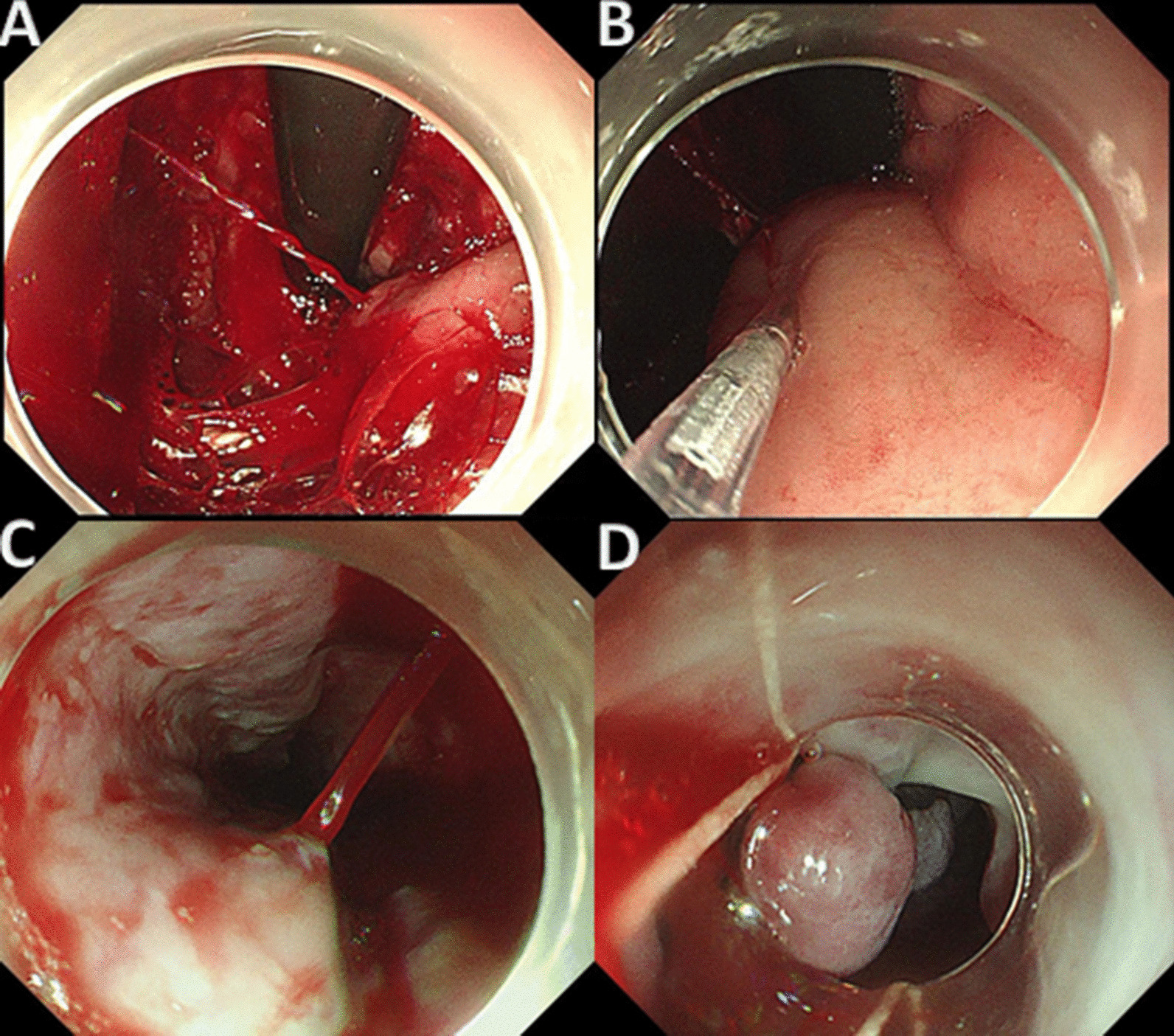
Endoscopic hemostasis techniques in patients with acute variceal bleeding. **A** and **B**: Endoscopic injection sclerotherapy and tissue adhesive is applied in the treatment of acute gastric variceal hemorrhage. **C** and **D**: Endoscopic variceal ligation is used in the treatment of acute esophageal variceal hemorrhage

Acute variceal bleeding (AVB) is a common and life-threatening complication of cirrhosis. Although there have been significant improvements in diagnostic and therapeutic modalities for the management of AVB in the recent several decades [1] [2], the mortality rate remains as high as 12–22% [3–5]. The mainstay of the management of patients with AVB includes resuscitation, pharmacological treatment (eg., Schistosoma and prophylactic antibiotics [6]), endoscopic treatment, etc. [7] Endoscopic procedures and endoscopic hemostasis techniques including endoscopic variceal ligation (EVL), Endoscopic injection sclerotherapy (EIS) and tissue adhesive (e.g. N-butyl-cyanoacrylate) are considered essential in the treatment of AVB [8, 9] (Fig. 1).

Current European consensus and American guidelines recommend emergency endoscopy to be performed within 12 h of admission [8–10], while Chinese guideline recommends emergency endoscopy to be performed 12–24 h after bleeding [9]. However, these recommendations are based on “expert opinion” and lack of solid evidence. There is a little related research to support these current recommendations. These researches were mostly retrospective and revealed contradictory results [11–15]. To date, a gold standard recommending the timing of endoscopy in patients with AVB has not been clearly determined.

This study aimed to explore the clinical outcomes in patients with AVB according to different timing to endoscopy. In addition, we aimed to investigate the clinical predictors of short-term mortalities, especially in patients at high risk for further bleeding or death.

## Methods

### Patients and study design

Medical records of esophagogastric varices bleeding patients undergoing endoscopy from our tertiary hospital were retrospectively reviewed between July 2014 and May 2022. The diagnosis of cirrhosis was established according to the previous history or on the combination of laboratory and imaging tests. The inclusion criteria were as follows: (1) Emergency patients admitted via the emergency room (ER) with upper gastrointestinal bleeding (UGIB); (2) Esophagogastric varices confirmed as the source of bleeding; (3) Endoscopy was performed within 24 h; (4) aged > 18 years. The exclusion criteria were as follows: (1) Severe dysfunction of a major organ (e.g., pulmonary disease, heart failure, and terminal malignancy except hepatocellular carcinoma); (2) UGIB from other than variceal bleeding (e.g., peptic ulcer bleeding); (3) Prior acute variceal bleeding (AVB) in the past three months; (4) Loss to follow-up within 6 weeks after AVB. This study protocol was approved by the Institutional Ethics Committee of Shanghai East Hospital and was performed in accordance with the Declaration of Helsinki.

### Interventions and data collection

When a patient with suspected AVB arrived at the ER, adequate fluid resuscitation, a vasoactive drug with somatostatin, and a prophylactic antibiotic were administered immediately. Packed red blood cell (PRBC) was given in the case of persisting bleeding or hemoglobin < 7 g/dl.

The endoscopy procedures were performed by five senior endoscopists with more than 3 years experience in the field of endoscopy. The timing of endoscopy depended on hemodynamic status, the discretion of the endoscopists on duty, and the patient’s will.

Endoscopic therapy was performed under the circumstances as follows: (a) Active bleeding of the varices; (b) Presence of adherent blood clot or the “white nipple” sign; (c) Presence of blood in the upper gastrointestinal tract and the varices as the only potential bleeding source; (d) In the case of the presence of varices without blood in the upper gastrointestinal tract, the endoscopist decided whether to perform the therapeutic endoscopy. The varices were treated by endoscopic band ligation (EBL), injection of a tissue adhesive (e.g., cyanoacrylate), sclerotherapy, or with the combination of hemoclips according to the discretion of the endoscopist.

Two independent investigators screened the medical records, including demographic, endoscopic reports, laboratory results, and clinical data. Model for End-stage Liver Disease (MELD) score [16], the Glasgow-Blatchford score (GBS) [17], Child-Turcotte-Pugh score (CTP) [18] were calculated.

### Follow-up and outcome assessment

The timing of endoscopy was defined as the interval between the hospital arrival and the initial endoscopic procedure, while the bleeding time was regarded as the duration between the beginning of the presence of bleeding and the initial endoscopic procedure. Endoscopy performed within 6 h of admission was defined as urgent endoscopy, and endoscopy performed within 6–24 h of admission was considered as early endoscopy.

The primary outcomes of this study were the 6-week mortality and re-bleeding rates. The secondary outcomes were the total number of blood units transfused, the length of hospital stay, and the need for salvage therapy (e.g., additional endoscopic therapy, balloon tamponade, transjugular intrahepatic portosystemic shunt [TIPS]).

### Statistical analysis

Means with standard deviations and frequencies with percentages were used for descriptive statistics. Statistical differences between the groups were investigated by the chi-square test and Fisher’s exact test for categorical variables and the Student’s t-test for continuous variables. Cumulative 6-week survival rates were estimated by Kaplan–Meier method and compared by log-rank test. Predictors of 6-week mortality were assessed by Cox’s proportional hazards model. A *P* value less than 0.05 was considered significant. Hazard ratios (HRs) and 95% confidence intervals (CIs) were also calculated. All statistical analyses were performed by SPSS version 20.0 (SPSS Inc., Chicago, IL, USA).

## Results

### Baseline characteristics

Overall, 352 AVB patients meeting the inclusion criteria from our tertiary center were admitted. Among them, according to the exclusion criteria, 40 patients were excluded. Finally, the remaining 312 patients were enrolled. Among them, 170 patients (54.49%) underwent urgent endoscopy, and 142 patients (45.51%) underwent early endoscopy.

As shown in Table 1, 195 (62.5%) patients were male, and the mean age was 62.98 ± 12.20. 142 (45.5%) patients had previous upper GI bleeding history. 61 (19.6%) patients had hepatocellular carcinoma (HCC). The most common etiology was chronic hepatitis B virus (HBV) infection, followed by AIH (autoimmune hepatitis) and alcohol use. The average time to endoscopy was 9.14 ± 8.70 h. The average Glasgow-Blatchford score, MELD score, and CTP were 11.22 ± 3.07, 14.21 ± 4.99, and 8.05 ± 1.69, respectively. 234 patients (75.0%) took beta blockers as prophylactic therapy for AVB before the bleeding episode. 138 patients (44.23%) were on anti-HBV specific treatment. 6 patients (1.92%) had portal vein tumor thrombus.

**Table 1:** Baseline characteristics of patients receiving urgent (< 6 h) versus early (6–24 h) endoscopy

Characteristics	Total (*N *= 312)	Urgent-endoscopy group (*N* = 170)	Early-endoscopy group (*N* = 142)	*P* value
Age (years)	62.98 ± 12.2	61.78 ± 13.74	64.42 ± 9.93	0.051
Male sex	195 (62.5%)	99 (58.2%)	96 (67.6%)	0.089
HCC	61 (19.6%)	29 (17.1%)	32 (22.5%)	0.225
*Etiology*				
HBV	225 (72.1%)	125 (73.5%)	100 (70.4%)	0.542
HCV	12 (3.8%)	6 (3.5%)	6 (4.2%)	–
AIH	29 (9.3%)	13 (7.6%)	16 (11.3%)	–
Alcohor	19 (6.1%)	8 (4.7%)	11 (7.7%)	–
Other	12 (24%)	21 (23.9%)	18 (23.9%)	–
*Coexisting diseases*				
Ischemic heart disease	53 (17%)	31 (18.2%)	22 (15.5%)	0.521
Cancer (except HCC)	35 (11.2%)	15 (8.8%)	20 (14.1%)	0.143
Diabetes mellitus	103 (33%)	49 (28.8%)	54 (38%)	0.085
High Blood Pressure	98 (31.4%)	57 (33.5%)	41 (28.9%)	0.378
COPD	25 (8%)	11 (6.5%)	14 (9.9%)	0.272
Prior upper GI bleeding	142 (45.5%)	82 (48.2%)	60 (42.3%)	0.291
Hepatic encephalopathy	24 (7.7%)	17 (10%)	7 (4.9%)	0.094
Ascites	142 (45.5%)	76 (44.7%)	66 (46.5%)	0.754
*Vital signs*				
Systolic blood pressure (mmHg)	112.92 ± 17.56	112.1 ± 18.31	113.89 ± 16.63	0.370
Heart rate (beat/min)	95.32 ± 16.19	96.71 ± 16.37	93.67 ± 15.88	0.099
*Prognostic scores*				
Glasgow-Blatchford score	11.22 ± 3.07	11.2 ± 3.02	11.24 ± 3.13	0.91
MELD score	14.21 ± 4.99	14.04 ± 4.92	14.42 ± 5.09	0.502
CTP score	8.05 ± 1.69	8.19 ± 1.83	7.89 ± 1.48	0.11
Time to endoscopy (hours)	9.14 ± 8.70	2.57 ± 1.40	17.03 ± 7.07	< 0.001
*Risk factors*				
Clopidogrel use	18 (5.8%)	11 (6.5%)	7 (4.9%)	0.561
Aspirin use	51 (16.3%)	30 (17.6%)	21 (14.8%)	0.497
Beta blockers	234 (75%)	129 (75.9%)	105 (73.9%)	0.694
*Laboratory*				
Hemoglobin	76.43 ± 21.10	76.16 ± 22.14	76.75 ± 19.85	0.807
Platelet (103/mm3)	100.21 ± 55.70	96.88 ± 58.31	104.20 ± 52.33	0.248
ALT	49.40 ± 72.47	48.21 ± 66.20	50.81 ± 79.54	0.753
Total bilirubin	38.01 ± 24.85	39.96 ± 26.83	35.68 ± 22.14	0.131
Serum albumin	28.48 ± 5.62	28.50 ± 6.60	28.45 ± 4.18	0.946
Prothromin time (INR)	1.37 ± 0.28	1.40 ± 0.29	1.34 ± 0.27	0.052
Serum creatinine	76.30 ± 70.48	73.66 ± 37.09	79.46 ± 96.37	0.5

*HCC* hepatocellular carcinoma, *HBV* hepatitis B virus, *HCV* hepatitis C virus, *AIH* autoimmune hepatitis, *COPD* Chronic Obstructive Pulmonary Disease, *GI* gastrointestinal, *MELD* model for end-stage liver disease, *CTP* Child-Turcotte-Pugh

The baseline patient characteristics, including age (*P* value = 0.051), gender (*P* value = 0.089), Glasgow-Blatchford score (*P* value = 0.91), CTP score (*P* value = 0.11), MELD scores (*P* value = 0.502), etc., did not differ significantly between the two groups. The median time to endoscopy was significantly different between the two groups (2.57 ± 1.40 vs. 17.03 ± 7.07; *P* < 0.001).

### Primary and secondary outcomes according to endoscopy timing

**Fig. 2:**
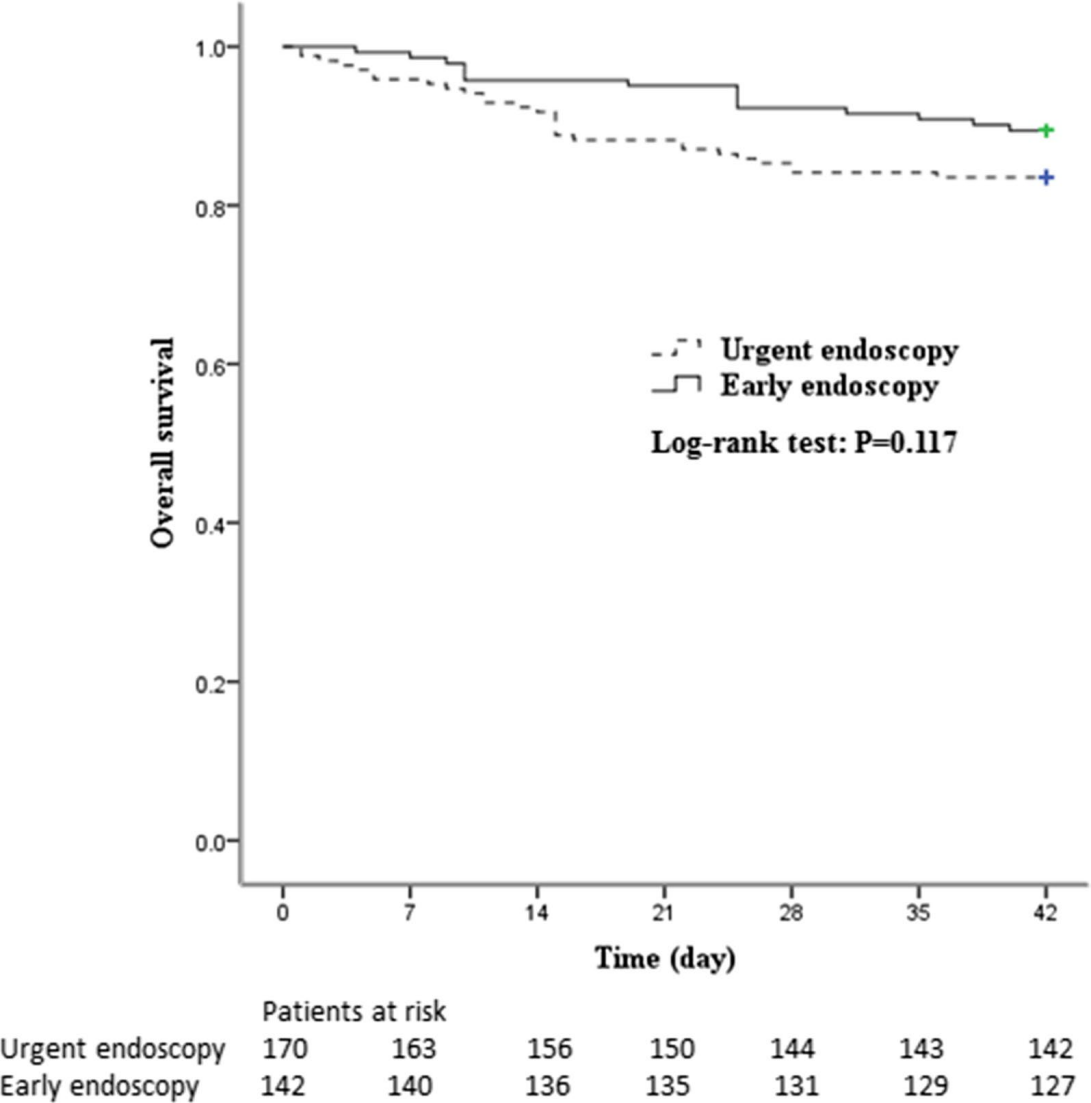
Comparison of six-week survival in the urgent endoscopy group and the early endoscopy group. The Kaplan–Meier survival plot is stratified by time to endoscopy of all patients, with solid line indicating early endoscopy (within 6 h) and the dotted line indicating urgent endoscopy (6–24 h)

The overall 6-week mortality rate was 13.8% (*n *= 43). Concerning the 6-week mortality, there were no significant differences between the urgent-endoscopy group and the early-endoscopy group (16.5% vs. 10.6%; *P* value = 0.117; Fig. 2). The overall 6-week re-bleeding rate was 13.5% (*n *= 42). There were no significant differences in the 6-week re-bleeding rate between the urgent-endoscopy group and the early-endoscopy group (11.2% vs. 16.2%; *P* value = 0.196). The overall 5-day re-bleeding rate was 4.49% (*n *= 14). The number of transfusions per patient (2.13 ± 3.17 vs. 1.4 ± 1.83; *P* value = 0.004) was significantly higher in the early-endoscopy group than in the urgent-endoscopy group. The length of hospital stay (15.1 ± 8.09 vs. 16.40 ± 7.88; *P* value = 0.153) and the rate of need for salvage therapy (5.9% vs. 3.5%; *P* value = 0.332) were not significantly different between the urgent-endoscopy group and the early-endoscopy group. (Table 2).

**Table 2:** primary and secondary outcomes

	Total (*N* = 312)	Urgent-endoscopy group (*N* = 170)	Early-endoscopy group (*N* = 142)	*P* value
6-week mortality	43 (13.8%)	28 (16.5%)	15 (10.6%)	0.132
6-week re-bleeding	42 (13.5%)	19 (11.2%)	23 (16.2%)	0.196
Number of blood units transfused	1.78 ± 2.55	1.40 ± 1.83	2.13 ± 3.17	0.031
Length of hospital stay (days)	15.69 ± 8.01	15.10 ± 8.09	16.40 ± 7.88	0.153
Need for salvage therapy	15 (4.8%)	10 (5.9%)	5 (3.5%)	0.332

### Predictive factors of 6-week mortality

By multivariate analysis, clinical predictors associated with 6-week mortality were explored, as shown in Table 3. Compared with early-endoscopy, urgent-endoscopy was not independently associated with short-term mortality (*P* = 0.345). However, the bleeding time shorter than 12 h (vs. more than 12 h) was independently associated with low short-term mortality (OR: 0.16; 95% CI: 0.06–0.46; *P* value = 0.001). The other independent risk factors of 6-week mortality were as follows: the presence of HCC (OR: 4.12; 95% CI: 1.94–8.71; *P* value < 0.001), the presence of encephalopathy (OR: 10.77; 95% CI: 4.35–26.62 *P* value < 0.001), high Glasgow-Blatchford score (OR: 1.14; 95% CI: 1.01–1.30; *P* value = 0.036) and high MELD score (OR: 1.15; 95% CI: 1.05–1.25; *P* value = 0.002).

**Table 3:** Multivariate analysis of predictors of 6-week mortality

	OR	*P* value
Time to endoscopy	–	0.345
Age	–	0.873
Gender	–	0.418
HCC	4.12 (1.94–8.71)	< 0.001
Beta blockers	–	0.346
Diabetes mellitus	–	0.574
Encephalopathy	10.77 (4.35–26.62)	< 0.001
Glasgow-Blatchford score	1.14 (1.01–1.30)	0.036
MELD score	1.15 (1.05–1.25)	0.002
CTP score	–	0.968
Re-bleeding within 5 days	–	0.971
Bleeding time (within 12 h)	0.16 (0.06–0.46)	0.001

*OR* odds ratio, *HCC* hepatocellular carcinoma, *HBV* hepatitis B virus, *HCV* hepatitis C virus, *MELD* model for end-stage liver disease, *CTP* Child-Turcotte-Pugh

In patients stratified by Glasgow-Blatchford score of 12 or higher (*n *= 138), multivariate analysis revealed HCC (OR: 2.56; 95% CI: 1.09–6.03; *P* value = 0.032) and encephalopathy (OR: 5.93; 95% CI: 2.22–15.89; *P* value < 0.001) as significant predictors of 6-week mortality. However, time to endoscopy was not associated with 6-week mortality (*P* value = 0.902). (Table 4).

**Table 4:** Multivariate analysis of predictors of 6-week mortality in patients with Glasgow-Blatchford score of 12 or higher

	OR	*P* value
Time to endoscopy	–	0.902
HCC	2.56 (1.09–6.03)	0.032
Encephalopathy	5.93 (2.22–15.89)	< 0.001
Bleeding time (within 12 h)	–	0.892

*OR* odds ratio, *HCC* hepatocellular carcinoma

## Discussion

The current study revealed that urgent endoscopy (< 6 h) is not significantly superior to early endoscopy (6–24 h) regarding the 6-week mortality or the 6-week re-bleeding rate. This phenomenon could be explained by the fact that basic resuscitation and medical treatments have a major influence on the patient’s outcome in the early stages of treatment [8, 10, 19]. Evidentially, if a patient undergoes an endoscopic procedure, basic resuscitation may be interfered during the critical early period of management, leading to a bad prognosis. Moreover, if endoscopy is performed too early, the quality of endoscopic examination may be suboptimal due to poor preparation. It may be marred by food residues and remnant blood clots. Thus, the procedure may be prolonged, and the risk of a procedure-related complication tends to increase [20, 21].

To explore the potential benefits of urgent endoscopy, we selected high-risk patients to perform the subgroup analysis. We applied the Glasgow–Blatchford score as a measure of risk, which has been shown to correlate with mortality [22]. An observational study revealed that a delay of endoscopy in high-risk non-variceal upper gastrointestinal bleeding (NVUGIB) patients with a Glasgow–Blatchford score of 12 or higher was correlated to a significant increase in mortality [23]. However, our study showed that urgent endoscopy remained unrelated to 6-week mortality in high-risk patients (Glasgow-Blatchford score ≥ 12).

To the best of our knowledge, five retrospective studies have investigated the optimal timing of endoscopic treatment in AVB patients. Two studies published in Taiwan in 2009 [11] and 2012 [13] showed that delayed endoscopy increased short-term re-bleeding and mortality. However, the technology and instruments in the field of endoscopy have been greatly developed in recent decades. The conclusion of these studies may not be representative of that of nowadays. Conversely, the Korean research published in 2019 [15] indicated that urgent endoscopy was significantly related to a poorer outcome in patients with AVB. Both the Canadian study published in 2009 [12] and the Korean study published in 2018 [14] revealed that the timing of endoscopy is independent of short-term mortality. A meta-analysis including the above five retrospective studies demonstrated that the time to endoscopy does not affect the mortality or re-bleeding rate of patients with AVB. However, there was significant heterogeneity in the process of analyses [24]. Our study also indicated that urgent endoscopy is not associated with short-term outcomes.

Most of the previous studies defined “urgent endoscopy” as within 12 h of admission. This study is one of the very few studies to compare the short-term outcomes in patients with AVB between urgent endoscopy (< 6 h) and early endoscopy (6–24 h) with a big sample size. Actually, it is difficult in many countries to perform urgent endoscopy within 6 h of admission. In the randomized controlled trial aiming to explore the timing of endoscopy for acute upper gastrointestinal bleeding, patients assigned to undergo endoscopy within 6 h were regarded as urgent-endoscopy group. However, in this urgent-endoscopy group, the time from presentation to gastroenterologic consultation was 7.4 ± 6.2 h; the time from gastroenterologic consultation to endoscopy was 2.5 ± 1.7 h; time from presentation to endoscopy was 9.9 ± 6.1 h [25]. In the present study, this limitation was overcome. Commonly, gastroenterologic consultation in our center was conducted within 1–2 h.

It is essential to select appropriate outcomes in studies of endoscopy. Since the long-term prognosis of patients with AVB is mainly associated with their basal liver function, it is inappropriate to demonstrate the precise effect of a single-point endoscopy by a long-term prognosis. Therefore, the endpoints were set to be the composite outcomes at 6 weeks in this study [8].

This is the first study to report the bleeding time (the duration between the start of bleeding and endoscopy), which might be more precise and valuable than the timing to endoscopy (the duration between the hospital arrival and endoscopy). Furthermore, we found that the bleeding time within 12 h was independently related to low 6-week mortality, while the timing to endoscopy was not associated with 6-week mortality. The inconsistency might be explained that basic resuscitation and medical treatments immediately after bleeding, other than timing to endoscopy, played an important role in short-term outcomes of patients with AVB. Endoscopy is a relatively invasive treatment, compared with other medical treatments. If unnecessary urgent endoscopies are frequently performed, medical staff fatigue and medical costs may increase dramatically [20]. Further researches are needed to explore the relationship between the bleeding time and short-term mortality of patients with AVB. This is also the first study to investigate the optimal timing to endoscopy in patients with AVB in the mainland of China. The main etiology of cirrhosis was HBV in our study, which was different from the baseline characteristics of the western countries, causing that the result of this study should be interpreted discreetly worldwide.

Despite these originalities, this study has some limitations. First, some bias may exist due to the retrospective nature of this study. Conducting patient randomization could address this limitation, but it would be problematic in these acutely ill patients because of their unstable vital signs and obvious ethical concerns. Second, the MELD score was recorded soon after the admission, but it might have changed after resuscitation and pharmacological treatment. Re-calculate the MELD score before the endoscopy might be more appropriate in predicting the risks. Third, those who had persistent hypotensive shock despite initial resuscitation were not included. Therefore, our trial findings should not be generalizable to AVB patients with persistent hypotensive shock. Forth, the Fibroscan value is not available in the present study, which might be useful in the multivariate analysis.

In conclusion, we found that the time to endoscopy does not appear to be associated with short-term mortality in patients with AVB and those who were at high risk for further bleeding or death. However, the bleeding time more than 12 h is an independent risk factor of high short-term mortality in patients with AVB. Our results should be taken into consideration when making clinical decisions. A multi-center prospective study with a bigger sample size from different countries is in demand to explore the optimal timing for endoscopy.

## Data Availability

The datasets used and/or analyzed during the current study are available from the corresponding author upon reasonable request.
